# Maternal and Prenatal Risk Factors for Childhood Leukemia in Southern of Iran

**Published:** 2011-06-01

**Authors:** J Hassanzadeh, R Mohammadi, A R Rajaeefard, M R Bordbar, M Karimi

**Affiliations:** 1 Department of Epidemiology, School of Health and Nutrition, Shiraz University of Medical Sciences, Shiraz, Iran; 2 Hematology Research Center, Shiraz University of Medical Sciences, Shiraz, Iran

**Keywords:** Leukemia, Children, Risk factors, Prenatal, Genetics

## Abstract

**Background:**

The causes of childhood leukemia as the most common malignancy in children are vastly unknown.The aim of this study is to evaluate the relationship between maternal birth characteristics with environmental exposures in childhood leukemia.

**Methods:**

This is a case-control study which consists of children younger than 18 years old suffering from leukemia who reside at Fars Province of Iran. Patients were individually matched with variables such as age, sex and residence region. In order to evaluate the relationships between each variable and the risk of leukemia, odds ratio(OR) and 95% confidence interval (CI) were estimated using conditional logistic regression.

**Results:**

Statistically, the association between risk of childhood leukemia with birth order (OR=6.177, 95%CI:2.551-14.957), pet ownership (OR=2.565, 95%CI: 1.352-4.868) and history of leukemia in first and second degree relatives (OR=2.667, 95%CI: 1.043-6.815) was significant. However, there was no significant association between daycare attendance, history of miscarriage, number of siblings and history of mother's diagnostic radiology tests with risk of childhood leukemia.

**Conclusion:**

Although no definite etiologic factor for acute childhood leukemia has been clearly defined, the contribution of environmental risk factors in the context of genetic predisposition are strongly elucidated.

## Introduction

Leukemia is the most common type of childhood malignancy which accounts for approximately 30% of cancer cases in children younger than 15 years old.[1][2][3] It is the primary cause of childhood cancer mortality in the United States and approximately 6000 new cases are diagnosed each year.[4] The etiology of childhood leukemia is mostly unknown. Epidemiologic studies have examined a number of possible risk factors including environmental, genetic and infectious causes. Links between most environmental risk factors (eg. electromagnetic fields, cigarette smoking) and childhood leukemia are inconsistent. However, researches present conclusive link between ionizing radiation and leukemia.[2][5]

Certain inherited diseases like fanconi anemia, bloom syndrome, ataxia telangiectasia, Schwachman syndrome and neurofibromatosis are associated with a higher risk of developing leukemia.[2] An identical twin is twice as likely as the general population to develop leukemia if the illness diagnosed before the age of 7 years.[6] Constitutional factors such as male gender, white race and high birth weight have been reported to increase the risk of leukemia.[7][8]

The infectious etiology hypothesis for childhood leukemia may be supported by socioeconomic and seasonal variations in the incidence of childhood leukemia as well as the clustering of leukemia cases.[9][10] Several indirect markers of exposure to infectious agents including birth order, daycare attendance and low socioeconomic class have been associated with leukemia.

Knowledge of these particular risk factors can be helpful in alleviating potentially harmful exposures and reducing the risk of diseases. The objective of this study was to examine the relationship between maternal, birth characteristics and environmental exposures with childhood leukemia.

## Materials and Methods

In this case-control study, children younger than 18 years old suffering from leukemia including acute lymphoblastic leukemia(ALL), acute myeloid leukemia(AML), and chronic myeloid leukemia (CML) who reside at Fars Province, Southern Iran were included. Their disease had been diagnosed by bone marrow aspiration and flowcytometry between 2005 to 2009 as they were undergoing chemotherapy at Pediatric Hematology-Oncology Ward in a referral Hospital, Shiraz, Southern Iran. The controls chosen by random sampling were individually matched to the cases by caliper matching method with respect to age (one year interval for children older than 2 years and 6 months interval for children younger than 2 years), sex and residence region. The information which was gathered through an interview with one of the parents included child's birth order, mother's age at the time of childbirth, child's birth interval from the last birth, number of siblings, history of miscarriage, history of leukemia in the immediate and secondary family members, history of mother's diagnostic radiography during pregnancy, child's day care attendance, father's occupation one year before child's birth, level of parents education, history of pet ownership and type of delivery. The birth weight of cases and control were recorded from their vaccination cards. Informed written consent was taken from the parents before entry to the study. In order to examine the relationship between each variable and risk of leukemia, odds ratio (OR) and 95% confidence interval (CI) were estimated using conditional logistic regression. Chi-Square test was used for data analysis. P value less than 0.05 was considered significant. The data were analyzed with SPSS software (version 15, Chicago, IL, USA).

## Results

Approximately 163 children younger than the age of 18 who suffered from leukemia were eligible for the study. The demographic characteristics of both cases and controls are shown in Table 1. There were no significant difference between the case and control groups regarding mother's age at the time of childbirth and level of parents' education. However, father's occupation one year before child's birth was significantly different in the two groups (p=0.004), particularly when agricultural working is concerned (Table 1).

**Table 1:** Demographic characteristics of cases and controls

**Variables**	**Cases frequency (%)**	**Controls frequency (%)**	**P value a**
Age (years)			
<4	51 (31.3)	51 (31.3)	Matched variable
5-9	54 (33.10	54 (33.10)	
10-14	39 (23.9)	39 (23.9)	
15-18	19 (11.7)	19 (11.7)	
Sex			
Male	100 (61.3)	100 (61.3)	Matched variable
Female	63 (38.7)	63 (38.7)	
Residency			
City	124 (76.1)	124 (76.1)	Matched variable
Village	39 (23.9)	39 (23.9)	
Maternal age (years)			
<20	19 (11.7)	29 (17.8)	P=0.057
20-24	47 (28.8)	48 (29.4)	
25-29	43 (26.4)	40 (24.5)	
30-34	35 (21.5)	39 (23.9)	
>35	19 (11.7)	7 (4.3)	
Total	163 (100)	163 (100)	
Maternal education			
Illiterate	21 (12.9)	11 (6.7)	P=0.080
Primary school	48 (29.4)	38 (23.3)	
Secondary school	36 (22.1)	38 (23.3)	
High school	44 (27)	50 (30.7)	
University degree	14 (8.6)	26 (16)	
Total	163 (100)	163 (100)	
Father education			
Illiterate	12 (7.4)	3 (1.8)	P=0.060
Primary school	39 (23.9)	32 (19.6)	
Secondary school	49 (30.1)	58 (35.6)	
High school	44 (27)	41 (25.2)	
University degree	19 (11.7)	29 (17.8)	
Total	163 (100)	163 (100)	
Father's occupation			
Employee	32 (19.6)	39 (23.9)	P=0.004
Self-employed	64 (39.3)	77 (47.2)	
Worker	35 (21.5)	37 (22.7)	
Agricultural worker	32 (19.6)	10 (6.1)	
Total	163 (100)	163 (100)	

^a^ Based on Chi-Square test

Table 2 shows the result of the estimated odds ratio by conditional logistic regression for various exposures. Although more cases than controls had high birth weight (>4000 g) (OR=2.431, 95%CI=0.60-9.861), this association was not statistically significant (p=0.214). After adjusting for maternal age and level of parental education, high birth order was associated with increased risk of childhood leukemia (OR=6.177, 95% CI=2.551-14.957, p<0.001).

**Table 2:** Estimated Odds ratio and 95% confidence interval through conditional logistic regression.

**Variables**	**subgroups**	**Case (N)/control(N)**	**Odds ratio**	**P value**	**Confidence interval**	
					**Low**	**High**
	<2500	11/12	1	-	-	-
Birth weight (g)^a^	2500-4000	121/144	0.929	0.837	0.374	2.303
	>4000	13/5	2.431	0.214	0.60	9.861
	1	63/96	1	-	-	-
Birth order^b^	2	40/36	1.768	0.094	0.907	3.448
	≥3	64/27	6.177	<0.001	2.551	14.957
Interval from last birth^b^	<2	27/20	1	-	-	-
	2-5	36/26	1.624	0.542	0.342	7.717
	<5	36/22	1.891	0.460	0.349	10.243
	<20	19/29	1	-	-	-
	20-24	47/48	1.190	0.671	0.534	2.653
Maternal age^c^	25-29	43/40	1.538	0.277	0.708	3.342
	30-34	35/39	1.073	0.872	0.454	2.539
	>35	19/6	3.041	0.076	0.890	10.358
	1	23/32	1		-	-
Sibship size^d^	2	60/78	1.471	0.638	0.571	3.787
	≥3	80/52	1.314	0.195	0.392	4.40
Pet ownership^e^		54/22	2.565	0.004	1.352	4.868
Day care attendance^f^		53/57	0.937	0.837	0.507	1.729
History of leukemia in relatives		16/6	2.667	0.040	1.043	6.815
History of mother's radiography		6/2	3	0.178	0.606	14.864
History of fetal loss^g^		22/16	1.211	0.590	0.603	2.435

^a^ Adjusted for maternal age and education,

^b^ Adjusted for maternal age, paternal education and maternal education,

^c^ Adjusted for paternal education, maternal education, paternal occupation,

^d^ Adjusted for maternal age, paternal education, paternal occupation, birth order,

^e^ Adjusted for paternal occupation,

^f^ Adjusted for level of maternal education, paternal occupation, birth order

^g^ Adjusted for maternal age.

More cases compared to controls (36 vs 22) had more than 5 years interval from the last birth, but this association was not statistically significant (p=0.46), neither maternal age over 35 years old (OR=3.041, 95% CI=0.89-10.385, p=0.076). Children with family history of leukemia were at a higher risk of developing this malignancy (OR= 2.667, 95% CI=1.043-6.815, p=0.040). Also, the research revealed significant association between pet ownership and risk of childhood leukemia (OR=2.565, 95% CI=1.352 – 4.868, p= 0.004).However, no significant association was identified between daycare attendance, miscarriage, number of siblings, and mother’s diagnostic radiography during pregnancy with increased risk of childhood leukemia (Table 2).

## Discussion

Cancers are assumed to be a multi-factorial disease which occur when the genetic and environmental factors interact in a multistage sequence. Leukemia as the most common childhood cancer is particularly subjected to this rule. Although, the recent increase in the childhood ALL cases may be partly explained by more accurate diagnostic practice and better reporting scheme, the role of new environmental risk factors must be more clearly elucidated.

The male predominance of leukemia cases in our study was previously reported by other researchers.[11][12] The peak occurrences of leukemia were in 5-9 years old children, which includes over sixty percent of children in this case. This is relatively compatible with the theory that young children (under 10 years of age) have less developed immune system and are more vulnerable to common childhood infections.

The role of infectious agents in developing leukemia was initially proposed by Greaves.[13] It was reemphasized by the population mixing theory of Kinlen,[14] and also the delayed infection mechanism introduced by Greaves and Alexander.[15] Moreover, seasonal variation in the birth or onset dates of childhood leukemia have been described which also supports the infectious etiology hypothesis.[16][17][18] In addition, increased risk of leukemia was noted in patients with a higher birth order (≥3), which may be explained by exposure to infectious agents at younger ages and in the populated families. Similar results were noted in other studies,[19][20][21] while some researchers have suggested a non-significant or even an inverse relationship.[2][4][5][22] The association between childhood leukemia and other markers of infection exposure including daycare attendance, and the family size was not proven in this study.

Several studies have described an association between increased birth weight and childhood ALL. Researchers believe it is due to the high rate of cell proliferation and risk of malignant transformation.[19][23][24][25] However, such association was not shown in our research, similar to what described by other investigators.[7][22]

In contrast to what Dockerty and colleagues described,[26] this study does not support existence of a link between advanced maternal age and risk of childhood leukemia. This could be partly explained by younger age of marriage and pregnancy especially in rural areas and the effect of genetics and other environmental factors. As stated earlier, we discovered significant difference between the case and control groups in regard to father's occupation a year before child's birth especially in agricultural working. This may be due to prolonged pesticide exposure or other unknown environmental hazards. However, there is inconclusive evidence regarding parent's occupational exposures and risk of developing leukemia. Results from the United Kingdom Child Cancer Study shows a small increased risk of childhood leukemia associated with parental exposure to exhaust fumes, and inhaled particulate hydrocarbons.[27] Other studies in Germany reported increase risks of childhood ALL in mothers exposed to paint or lacquer during the preconception period.[28] In 2001, Cordier conducted a multi-center case-control study, which exhibited an increased risk of childhood brain tumors in farmer families.[29] As most studies are generally performed by self-administered questionnaires, assessing chemical environmental links to childhood leukemia, and parent’s occupational exposure is not obviously relevant, thus remains elucidated.

Bross and Gibson reported in 1970, that the exposure to pets could be a factor of the development of leukemia. They discovered the increase risk of leukemia among children exposed to sick cats to two-folds.[30] This was also confirmed by Petridou et al. (1997) in Greece. However, Swenson and colleagues proved to be exactly opposite.[31] The concern that pets may be associated with childhood leukemia arose when oncogenic viruses such as Feline Leukemia Virus (FELV) were discovered in animals.[30][32]

Based on our findings, there is a positive significant link between the history of leukemia among first and second degree relatives, comparable to Ripert's study.[33] Siblings of children with this acute/chronic disease are at a greater risk of developing leukemia than the general population. The importance of inutero genetic events has been suspected for many years based on concordance studies on twins with leukemia. Some leukemogenic translocations and other markers of clonality can be detected at birth in hematopoietic cell population. Although the majority of these children do not go on to develop overt leukemia, environmental factors, viral infection, and immunodeficiency states may provoke these genetically predisposed children to leukemia.

Child miscarriage and risk of childhood leukemia have been pursued in several studies and have reached contradictory results. While some studies similar to this study did not show any significant associations,[7][19] others suggest it as a possible risk factor.[4][20][34] These controversies may be explained as follows: First, the characteristics of leukemia cases in different studies varied. Second, such information was gathered through interviews in some studies which may be subjected to recall biases.

There are inconsistent results regarding fetal irradiation from prenatal exposure to diagnostic radiographs and risk of childhood leukemia. While Doll and Wakeford estimated a relatively 6% increased risk of cancer per GY,35 results from other studies like ours have not supported this association.[36][37][38] However, due to small sample size and limitations imposed by recall biases, the results should be interpreted cautiously.

In this study, we attempted to follow a comparable accuracy principle by informality in data gathering in both case and control groups. However, like other case-control studies, our research has been subjected to error due to biased information, especially in regard to variables dependent to recall.

As with much other human diseases, both genetic and environmental factors have been implicated in the etiology of childhood leukemia. Data to support the exact causative factors are often inconsistent and somehow contradictory. This study presented an existence of a link between increased birth order, pet ownership and history of leukemia among first and second degree relatives to be significant. The contradiction presented in different studies regarding different variables is partly explained by the influence of genetic predisposition and leukomogenic translocations. Furthermore, the multi-center matched case-control studies are recommended to clarify the ambiguous aspects of this complex subject.
